# The Malingering Intussusception

**DOI:** 10.5811/cpcem.2017.3.33793

**Published:** 2017-10-03

**Authors:** Jacqueline Le, Joel Labha, Babak Khazaeni

**Affiliations:** * Desert Regional Medical Center, Department of Emergency Medicine, Palm Springs, California; † Arrowhead Regional Medical Center, Department of Emergency Medicine, Colton, California

## Abstract

While intussusception is rarely seen in adults, it is typically obstructive in nature when it does occur. Even less commonly seen is transient intussusception, which occurs without a radiological lead point or any evidence of bowel obstruction. Such findings consist of a “target pattern” seen on computed tomography (CT) but are incidental and do not require any surgical intervention. We report the case of a 31-year-old female who presented to the emergency department with abdominal pain, vomiting, and diarrhea. CT imaging revealed transient intussusception, a benign finding that is not well established in emergency medicine literature.

## INTRODUCTION

In emergency medicine (EM), abdominal pain is a common complaint. The etiology of abdominal pain is vast and we are constantly faced with multiple diagnostic challenges. This case highlights a presentation of abdominal pain with a diagnostic imaging dilemma: a relatively benign exam with an unusual finding on computed tomography (CT). Intussusception, often considered a surgical emergency, is a common pediatric diagnosis but rarely seen in adults. We discuss the case of an incidental finding of adult intussusception on CT imaging. With the increasing use of CT in patients with abdominal pain, we are likely to see more of this transient and benign finding in the emergency department (ED). Although it has been discussed in surgical and radiological literature,^1^,^6^–^9^ it is still a comparatively unfamiliar entity in EM literature, thereby motivating this case discussion.

## CASE REPORT

A 31-year-old Caucasian female with history significant for chronic recurrent pancreatitis, endometriosis, anxiety, depression, and previous cholecystectomy presented to the ED with abdominal pain for two days. She described the pain as constant, stabbing, and localized to both lower quadrants without radiation. She also complained of non-bloody, bilious emesis “too numerous to count” with non-bloody diarrhea. She denied any fever, dysuria, or vaginal bleeding or discharge. On presentation, her vital signs were normal but she appeared anxious and in moderate distress. Her abdominal examination revealed a soft, non-distended abdomen with normoactive bowel sounds. She was diffusely tender to palpation without rebound or guarding. There was no palpable mass, evidence of McBurney’s point tenderness, or Rovsing’s sign. The remainder of her physical examination was unremarkable.

Review of the patient’s laboratory tests, including complete blood count, basic metabolic panel, liver function tests, lipase, and urinalysis revealed no significant abnormalities. Human chorionic gonadotropin urine test was negative. The patient was given intravenous normal saline, ketorolac, ondansetron, and lorazepam for symptomatic control but she later noted only mild pain relief. A contrast-enhanced CT of the abdomen and pelvis was obtained and showed normal kidneys, pancreas, and appendix. There was no free air, free fluid, biliary dilatation, or pericolic inflammatory change. Stool was present in the right colon with fluid in the small bowel representing mild constipation. An incidental finding of a jejunal short segment intussusception in the left upper quadrant was seen without any evidence of bowel obstruction (see Image). Following discussion with the radiologist, it was determined to be a benign finding and completely asymptomatic in the absence of a small bowel obstruction. The patient was subsequently witnessed by nursing staff to induce vomiting while specifically requesting hydromorphone. The patient appeared comfortable and in no acute distress on multiple occasions while not being directly observed; however, when approached she promptly complained of unrelenting pain of 10 out of 10 severity. The patient was medicated with intravenous hydromorphone and shortly thereafter reported complete resolution of her abdominal pain and nausea. She subsequently admitted to multiple other gastrointestinal (GI) workups at other EDs and with her primary care physician (PCP) with no abnormalities found other than chronic pancreatitis.

## DISCUSSION

Intussusception, the telescoping of one portion of the intestine into a contiguous segment, is a clinical entity that has been well described in children. It is a common cause of abdominal pain in the pediatric population and is usually idiopathic. However, intestinal intussusception is rare in adults, accounting for just 5% of all intussusceptions.^2^ With considerable variability, the symptoms of adult intussusception are broad; rarely seen is the classic triad of abdominal pain, a tender palpable mass, and bloody stools. Instead, the symptoms of vomiting, GI bleed, constipation, or abdominal distention are seen.^2^ The most common presentation in adults is intermittent abdominal pain, but this has been described in cases of intussusception caused by an organic lead point such as a mass or lesion that led to the intussusception and subsequently a mechanical small bowel obstruction.^1^,^3^ In one series, two cases of idiopathic adult jejunal intussusceptions were diagnosed on CT after both patients presented with nonspecific abdominal pain and nausea; neither of them required surgical intervention and no underlying abnormality or lead point was found.^4^

**CPC-EM Capsule**

What do we already know about this clinical entity?

Intussusception is the telescoping of one segment of the intestine into an adjoining section. It is the most common surgical emergency in the pediatric population but a rare occurrence in adults.

What makes this presentation of disease reportable?

Unlike classic intussusception, transient intussusception is visualized on computed tomography (CT) as a “target pattern” without bowel obstruction and requires no further management.

What is the major learning point?

Adult transient intussusception is an incidental radiographic finding and should not prompt any surgical intervention.

How might this improve emergency medicine practice?

With the increasing use of CT imaging in patients with abdominal pain, knowledge of this benign and transient finding will lead to timely recognition and disposition.

In the absence of an inciting factor such as an organic lesion as in this case, a transient non-obstructing intussusception without a lead point was identified. Although most often idiopathic, this type of intussusception has been seen in some patients with celiac or Crohn’s disease. It does not require surgical intervention and will resolve on its own.^2^ On the other hand, classic intussusception with a lead point typically involves an obstruction and has been attributed to conditions such as inflammatory bowel disease, adhesions, malignancy, and trauma.^1^,^2^ In this case, the discrepancy between the locations of her pain and the intussusception, a benign physical examination, normal laboratory results, no CT evidence of obstruction, and the patient’s possible malingering behavior all support that the intussusception was nothing more than an incidental finding.

Despite being operator dependent, ultrasound is currently considered the imaging diagnostic modality of choice in children.^5^ However, the most sensitive test in adults is the CT, with sensitivities between 58–100%.^1^,^2^ Transient non-obstructing intussusception in adults has been discussed in the radiological literature^6^–^9^ but is not commonly recognized in EM, thereby prompting this case discussion. We further investigated what CT findings would more likely represent a transient intussusception as opposed to an intussusception requiring either medical or surgical intervention. The features seen on CT that help distinguish transient intussusception from obstructing intussusception include a “short…soft tissue density structure extending into the bowel lumen,” “triangular or crescent-shaped fat density due to the eccentrically placed mesentery,” and “normal calib[er] of the involved loop….[and] loops proximal to the intussusception.”^6^ CT evidence of the classically described “target pattern,” as seen in this case, corresponds to an “initial intussusception” without any signs of ischemia.^1^,^6^ The progressive grades of obstruction seen on CT imaging correspond to what is described as a “reniform pattern” and then as a “sausage-shaped pattern” representing the last stage of the disease.^6^

In this case, the classic “target pattern” was clearly visualized on the coronal sections of the CT images of the abdomen and pelvis, and the patient was monitored in the ED with complete resolution of her symptoms. On reevaluation, abdominal examination revealed normoactive bowel sounds and a non-distended, soft abdomen without any tenderness to palpation. There were no palpable masses or evidence of rigidity, rebound, or guarding. Coupled with the patient’s clinical presentation and our discussion with the radiologist, the patient was discharged home and instructed to follow up with her PCP and established gastroenterologist. A follow-up telephone call was attempted five days after discharge; however, the contact phone number provided by the patient was found to be invalid.

Review of her chart later revealed five additional ED visits also for abdominal pain. The first of these five other visits occurred only two months after her initial presentation. Two subsequent CTs performed on her did not demonstrate any target pattern, bowel obstruction, or other acute abnormality. She was discharged home in improved condition on all visits. These ensuing visits and CT images further support the transient nature of the intussusception seen initially.

## CONCLUSION

In conclusion, this case demonstrates a unique finding not well documented in the EM literature. Unlike obstructive intussusception with a lead point, transient non-obstructing intussusception can present as an incidental finding that should not prompt emergent surgical evaluation in the ED.

## Figures and Tables

**Image AB:**
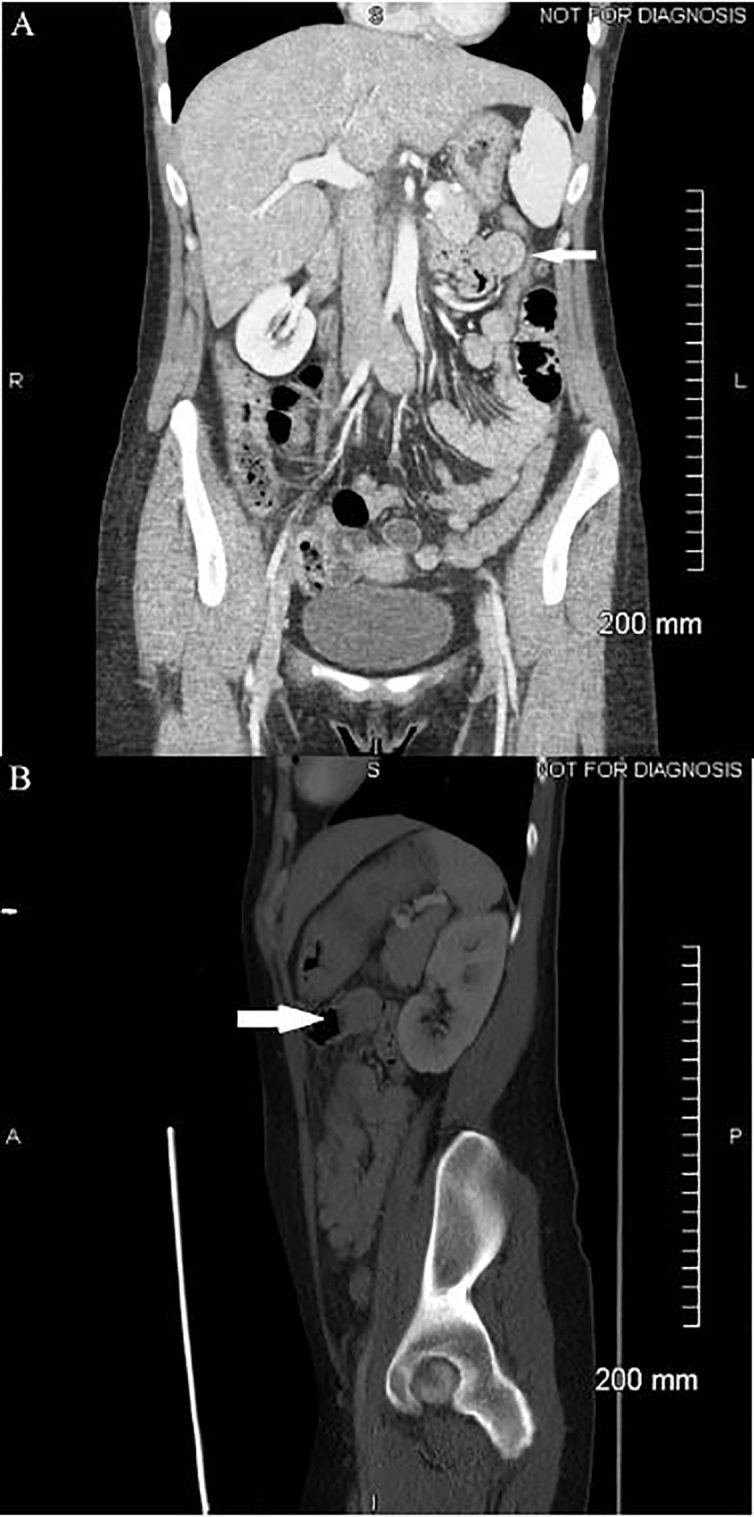
Computed tomography (CT) images of the abdomen and pelvis demonstrating transient, non-obstructive jejunal intussusception: (A) Coronal view depicting the classic “target pattern” (small arrow) in the left upper quadrant; (B) Saggital view showing the jejunal short segment intussusception (big arrow) without any evidence of bowel obstruction.
